# Effects of acidic and neutral fluoride gels on surface microhardness and morphology of composite resins used in pediatric dentistry: an in vitro factorial study

**DOI:** 10.1186/s12903-026-08545-7

**Published:** 2026-05-08

**Authors:** Eser Rengin Nalbantoglu, Oyun-Erdene Batgerel, Rafat Sasany, Yesim Ayla, Rawan Aldeijani, Zuhre Hale Cimilli

**Affiliations:** 1 Department of Pediatric Dentistry, Faculty of Dentistry, Biruni University, Merkezefendi Mahallesi, G/75. Sokak No:13B, Zeytinburnu, Istanbul 34015 Turkey; 2 Department of Restorative Dentistry, Faculty of Dentistry, Biruni University, Merkezefendi Mahallesi, G/75. Sokak No:13B, Zeytinburnu, Istanbul 34015 Turkey; 3 Department of Prosthodontics, Faculty of Dentistry, Biruni University, Merkezefendi Mahallesi, G/75. Sokak No:13B, Zeytinburnu, Istanbul 34015 Turkey; 4 Cekmekoy Oral and Dental Health Center, TC Ministry of Health, Cekmekoy, Istanbul 34854 Turkey; 5 Paediatric Dentistry Unit, Jaber Al-Ahmad Specialized Dental Center, Ministry of Health, Jamal Abdel Naser Street, Sulaibkhat, Kuwait; 6 Department of Endodontics, Faculty of Dentistry, Marmara University, Basibuyuk Yolu 9/3 Maltepe, Istanbul, 34854 Turkey

**Keywords:** Composite resins; Topical fluoride, Surface microhardness, Acidulated phosphate fluoride, Sodium fluoride, Pediatric dentistry, Scanning electron microscopy

## Abstract

**Background:**

Concerns persist regarding the effects of repeated topical fluoride application on the surface integrity of composite resins. This study specifically evaluated how acidic and neutral fluoride formulations and varying exposure durations influence the surface microhardness and morphology of pediatric dental composite resins.

**Methods:**

Four light-cured composite resins (Filtek Z250, FSM Hi-Fill Kids Blue, FSM Hi-Fill Kids Yellow, and Gænial) were evaluated. Specimens were exposed to acidulated phosphate fluoride (APF) or neutral sodium fluoride (NaF) gels for either 4–24 h to simulate short-term and repeated exposure conditions. Surface microhardness was assessed using a Vickers microhardness tester (100 g load, 15 s dwell time), and surface morphology was examined qualitatively using scanning electron microscopy (SEM). Data were analyzed using three-way ANOVA to evaluate the effects of composite type, fluoride type, exposure duration, and their interactions.

**Results:**

Composite type and exposure duration significantly influenced surface microhardness (*p* < 0.05), whereas the main effect of fluoride type was not statistically significant. However, significant interaction effects indicated that the response to fluoride exposure was material-dependent. Overall, APF exposure resulted in greater reductions in microhardness and more pronounced surface changes compared with NaF, particularly after prolonged exposure. Filtek Z250 consistently exhibited the highest microhardness values, whereas Gænial showed the lowest values across most experimental conditions. SEM findings supported these results, revealing more evident surface irregularities and matrix alterations following APF exposure.

**Conclusions:**

Topical fluoride applications affected the surface microhardness of composite resins in a material-dependent manner. Acidulated fluoride formulations induced greater surface alterations than neutral formulations, particularly under prolonged exposure conditions. From a clinical perspective, neutral fluoride formulations may be preferred over acidic agents to minimize surface degradation and preserve the longevity of composite restorations in pediatric patients receiving repeated preventive care.

## Background

Dental composite resins are widely used in restorative dentistry because of their favorable esthetic properties, adhesive capability, and continuous improvements in mechanical performance [1, 2]. In pediatric dentistry, composite restorations are frequently preferred for primary and young permanent teeth, where minimal intervention and preservation of tooth structure are essential. However, the long-term durability of composite resins in the oral environment may be compromised by chemical challenges arising from oral fluids and preventive agents, including topical fluoride formulations [2, 3]. These processes are largely related to hydrolytic degradation of the resin matrix and weakening of the filler–matrix interface within polymer networks exposed to aqueous and acidic environments [4].

Despite their widespread use, topical fluoride agents have been shown to interact with resin-based restorative materials, although the extent of these effects may vary depending on material composition and exposure conditions [5, 6]. Fluoride exposure may reduce surface hardness through chemical softening of composite resins, particularly under acidic conditions that promote resin matrix degradation and weaken the filler–matrix interface [3, 7]. Acidulated phosphate fluoride (APF) gels enhance fluoride uptake due to their low pH; however, they may also promote surface alterations in resin-based composites, including increased roughness and surface irregularities [7, 8]. Neutral sodium fluoride (NaF) gels are generally considered less aggressive, with studies reporting less pronounced surface alterations compared with acidic fluoride formulations [8].

Surface microhardness indicates the resistance of restorative materials to wear, deformation, and chemical degradation. Changes in microhardness signal softening and structural alteration due to environmental stress [9]. Clinically, reduced microhardness can increase wear, reduce polish, and raise plaque retention, compromising restoration longevity. In vitro studies have shown that the pH and composition of fluoride formulations can affect composite resin surfaces, with acidic gels typically producing more pronounced changes than neutral formulations [7, 10]. However, reported effects vary due to differences in composite formulation, fluoride type, concentration, and exposure [7, 10].

Surface microhardness indicates resistance of restorative materials to wear and degradation. Changes in microhardness signal softening and structural alteration due to environmental stress [9]. Clinically, lower microhardness can increase wear, reduce polish, and raise plaque retention, compromising restoration longevity. In vitro studies show that the pH and composition of fluoride formulations can affect composite resin surfaces—acidic gels typically cause greater changes than neutral ones [7, 10]. However, reported effects vary due to differences in composite makeup, fluoride type, concentration, and exposure [7, 10].

In pediatric dental practice, preventive care commonly includes routine fluoride applications, particularly in children at high risk of dental caries, while composite resin restorations are widely used in both primary and young permanent dentitions. As a result, restorative materials are repeatedly exposed to fluoride agents throughout their clinical lifespan. Although the anticaries benefits of fluoride are well established, the potential cumulative effects of repeated fluoride exposure on the surface integrity of composite restorations remain insufficiently understood. From a clinical perspective, maintaining both the preventive efficacy of fluoride and the structural integrity of restorative materials is essential for optimizing long-term treatment outcomes in pediatric patients.

Importantly, comparative data remain limited, particularly for contemporary composite systems used in pediatric dentistry under varying fluoride exposure conditions, and evidence on the combined influence of fluoride type and exposure duration on surface properties is still scarce. Given the cumulative nature of fluoride exposure over time, prolonged exposure protocols can serve as accelerated models for investigating material behavior rather than simulating a single clinical application. The materials included in this study were selected to represent commonly used composite systems in pediatric dentistry with differing filler characteristics and resin matrix compositions, which may influence their resistance to chemical degradation. Therefore, this study aimed to evaluate, under controlled in vitro conditions, the effects of acidic and neutral topical fluoride applications and exposure duration on the surface microhardness and SEM-assessed morphology of commonly used composite resins in pediatric dentistry.

## Methods

### Study design

This in vitro study evaluated the effects of composite type, treatment condition, and exposure duration on the surface microhardness and surface morphology of four light-cured composite resin materials. The experimental design followed a factorial arrangement based on composite material (4 levels), treatment condition (APF, NaF, and distilled water [control]), and exposure duration (4 min and 24 h).

Accordingly, 16 fluoride-treated experimental groups were established (4 composites × 2 fluoride gels × 2 exposure durations), with 10 specimens per group (*n* = 10), yielding 160 fluoride-treated specimens for microhardness testing. In addition, for each experimental condition, 10 time-matched control specimens were immersed in distilled water for the corresponding exposure duration (4–24 h) and processed identically thereafter. Thus, an additional 160 control specimens were included, resulting in a total of 320 specimens allocated for microhardness analysis.

Separately, additional specimens were fabricated for SEM evaluation, with two specimens prepared for each fluoride-treated experimental group and two for each corresponding control group. Consequently, 32 fluoride-treated specimens and 32 control specimens were examined by SEM (total SEM *n* = 64). Overall, 384 specimens were prepared in the study.

### Composite resin materials

The composite resins evaluated in this study were selected based on their relevance to contemporary pediatric restorative dentistry. Among these materials, Hi-Fill Kids may be considered a pediatric-specific composite resin, as its colored formulations are designed to enhance child engagement and facilitate restorative procedures. The remaining materials, although not specifically developed for pediatric use, are commonly used in the restoration of primary and young permanent teeth due to their favorable mechanical properties and clinical versatility. Therefore, these materials were selected to represent a range of composite systems relevant to pediatric clinical practice. A total of four light-cured composite resin materials were evaluated in this study. Detailed information regarding the manufacturer, country of origin, composite classification, and filler content is provided in Table 1.

**Table 1:** Characteristics of the composite resin materials evaluated in the study

Composite material (Color)	Manufacturer	Country	Composite type	Filler content*
Filtek Z250 (A2)	3 M ESPE	St. Paul, MN, USA	Microhybrid composite	82% wt (68% vol)
FSM Hi-Fill Kids (Blue)	FSM Dental	Ankara, Turkey	Nanohybrid composite	70% wt (67% vol)
FSM Hi-Fill Kids (Yellow)	FSM Dental	Ankara, Turkey	Nanohybrid composite	70% wt (67% vol)
Gænial (A2)	GC Corporation	Tokyo, Japan	Nanohybrid composite	73% wt (64% vol)

*According to manufacturer information

### Fluoride agents and application protocol

Two topical fluoride gels were used: a NaF gel (Mirafluor-ID; 2% NaF, pH 7; Hager & Werken, Duisburg, Germany) and an APF gel (Ionite; 1.23% APF, pH 3–4; USA). For the fluoride-treated groups, specimens from each composite material were randomly assigned to either the APF or NaF condition and exposed using either a short-term (4 min) or a prolonged (24 h) protocol.

For the 4-min protocol, each specimen was placed individually in a 24-well microplate and immersed in the assigned fluoride gel for 4 min, simulating professional chairside application. After exposure, specimens were thoroughly rinsed with distilled water and gently air-dried. For the prolonged protocol, specimens were exposed to the assigned fluoride gel for a total of 24 h using an intermittent regimen over 3 consecutive days (8 h/day), as previously described in a home-use simulation study [11]. Each day, specimens were removed from distilled water, gently blotted dry, placed individually into 24-well microplates, and covered with fluoride gel to ensure complete immersion and continuous surface contact during the 8-h exposure period. To minimize local chemical equilibrium at the specimen surface, the plates were gently agitated at hourly intervals. After each daily exposure period, specimens were thoroughly rinsed with distilled water and stored in distilled water storage until the next cycle. This prolonged exposure model was used as an accelerated protocol to investigate the potential cumulative effects of repeated fluoride contact on composite resin surfaces, rather than to simulate a single clinical application. According to Dionysopoulos et al. [11], a total exposure time of 24 h may approximate 1 year of daily 4-min fluoride application in an accelerated in vitro model. Time-matched control specimens underwent the same protocol using distilled water, with exposure durations of either 4 min or 3 × 8 h and were processed identically thereafter.

### Specimen preparation

For each composite material, disc-shaped specimens were fabricated using a custom-made stainless-steel bipartite mold (8 mm in diameter and 2 mm in thickness) to ensure standardized dimensions [12]. Standardization of specimen geometry was performed to minimize inter-specimen variability and improve the reliability of subsequent microhardness and morphological analyses [6, 13]. Composite resin was inserted into the mold in a single increment to ensure complete filling and minimize void formation [8]. Specimens were prepared using Mylar strips to obtain standardized flat surfaces and were light-cured for 20 s with a calibrated LED unit (1000 mW/cm²) under standardized conditions [14, 15].

After polymerization, the specimens were removed from the mold and stored in distilled water at 37 °C for 24 h before the experimental procedures to allow post-polymerization maturation and standardization of baseline material properties [16–18]. The specimens were then randomly allocated to their respective experimental groups [19–21].

### Surface microhardness testing

Surface microhardness was measured using a Vickers microhardness tester (Wilson Tukon 1102, Buehler, USA). A load of 100 g was applied for 15 s for each indentation. Vickers hardness (HV) values were recorded from the central region of each specimen surface to minimize potential edge effects. The HV value obtained from each specimen was used to calculate the mean surface microhardness for each experimental group (*n* = 10) for subsequent statistical analysis. All measurements were performed by a single calibrated examiner who was blinded to the experimental groups. Intra-examiner reliability was assessed by repeated measurements on a subset of specimens.

### Scanning electron microscopy (SEM) analysis

Qualitative surface morphology was assessed using SEM. Two specimens from each fluoride-treated experimental group and two specimens from each corresponding control group were selected as representative samples for SEM evaluation (*n* = 2 per group). These specimens were selected to provide a representative and exploratory visualization of surface morphology rather than quantitative analysis. The specimens were sputter-coated with gold–palladium (Au/Pd) using a SC7620 sputter coater (Quorum Technologies, UK) at 18 mA for 120 s to improve surface conductivity. Surface morphology was examined using a scanning electron microscope (Zeiss EVO MA10, Zeiss, Germany) under high-vacuum conditions at an accelerating voltage of 10 kV with a secondary electron detector. Images were acquired at magnifications of ×500 and ×2000.

### Statistical analysis

A priori power analysis was conducted to determine the minimum sample size required for the study. With a significance level of α = 0.05, a statistical power of 1 − β = 0.80, and an expected effect size of d = 0.5, the minimum sample size was calculated as 10 specimens per group. Statistical analyses were performed using IBM SPSS Statistics (version 20.0; IBM Corp., Armonk, NY, USA). Descriptive statistics were expressed as mean ± standard deviation (SD). Data normality was assessed using the Kolmogorov–Smirnov test together with visual examination of histograms and Q–Q plots.

Surface microhardness values were analyzed using a three-way analysis of variance (ANOVA) to assess the effects of composite type, treatment condition, and exposure duration, along with their interaction terms. The composite type included four levels, the treatment condition included three levels (APF, NaF, and distilled water control), and the exposure duration included two levels (4 min and 24 h). When the ANOVA revealed statistically significant differences, Tukey’s honestly significant difference (HSD) post hoc test was used for pairwise comparisons. Statistical significance was established at *p* < 0.05.

## Results

### Surface microhardness

Surface microhardness values (Vickers hardness, HV) are presented in Table 2. Composite material had a significant effect on microhardness (*p* < 0.001), with Filtek Z250 consistently exhibiting the highest values and Gænial the lowest across most conditions. Exposure duration also significantly influenced microhardness (*p* < 0.05), whereas the main effect of treatment condition was not statistically significant.

**Table 2:** Surface microhardness (HV) values of the composite resins under different treatment conditions and exposure durations (*n* = 10)

Material	Control 4-min	APF 4-min	NaF 4-min	Control 24-h	APF 24-h	NaF 24-h
	Mean ± SD (HV)	Mean ± SD (HV)	Mean ± SD (HV)	Mean ± SD (HV)	Mean ± SD (HV)	Mean ± SD (HV)
Filtek Z250	78.63 ± 2.57	78.29 ± 2.35^a^	80.95 ± 2.51^a^	75.74 ± 0.64	81.58 ± 3.44^a^	80.49 ± 5.34^a^
FSM Hi-Fill Kids (Blue)	51.69 ± 1.38	51.80 ± 1.63^b^	47.58 ± 2.98^b^	57.04 ± 0.66	49.47 ± 4.65^b^	48.16 ± 7.38^bc^
FSM Hi-Fill Kids (Yellow)	48.08 ± 2.55	46.84 ± 6.25^c^	47.58 ± 5.15^b^	53.17 ± 1.64	48.42 ± 7.22^b^	50.23 ± 7.51^b^
Gænial	33.81 ± 0.76	32.70 ± 3.69^d^	34.59 ± 3.78^c^	37.20 ± 0.73	37.13 ± 2.51^c^	42.39 ± 5.21^c^

Values are presented as mean ± standard deviation in Vickers hardness (HV). Different superscript letters indicate statistically significant differences between composite materials within each treatment condition and exposure duration (Tukey HSD, *p* < 0.05)

Significant interaction effects were observed between composite material and treatment condition, and between composite material and exposure duration. A significant three-way interaction among all factors was also detected, while the interaction between treatment condition and exposure duration was not significant. These findings indicate that the effect of fluoride exposure on microhardness depended on both material type and exposure duration. Although the main effect of treatment condition was not statistically significant, the greater reductions in microhardness observed in APF-treated groups under prolonged exposure are attributable to interactions among fluoride type, material, and exposure duration rather than to fluoride type alone.

Across both 4-min and 24-h exposures, Filtek Z250 maintained the highest hardness values, whereas Gænial consistently demonstrated the lowest. FSM Hi-Fill Kids (Blue and Yellow) showed intermediate values. Control specimens exhibited relatively stable hardness over time.

**Fig. 1:**
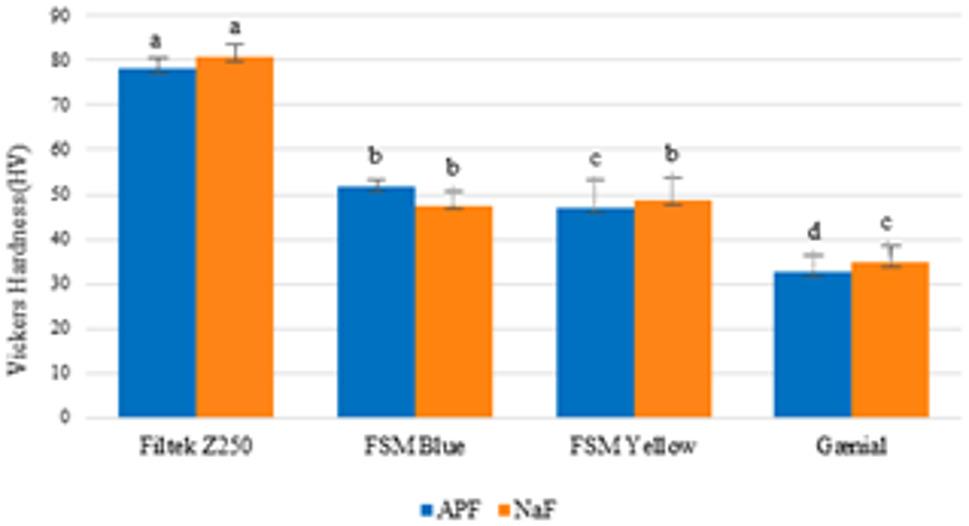
Comparison of surface microhardness (mean ± SD, HV) of composite resins after 4-minute exposure to APF and NaF gels. Bars represent mean values, and error bars indicate standard deviation. Different superscript letters indicate statistically significant differences between materials within the same condition (Tukey HSD, *p* < 0.05)

**Fig. 2:**
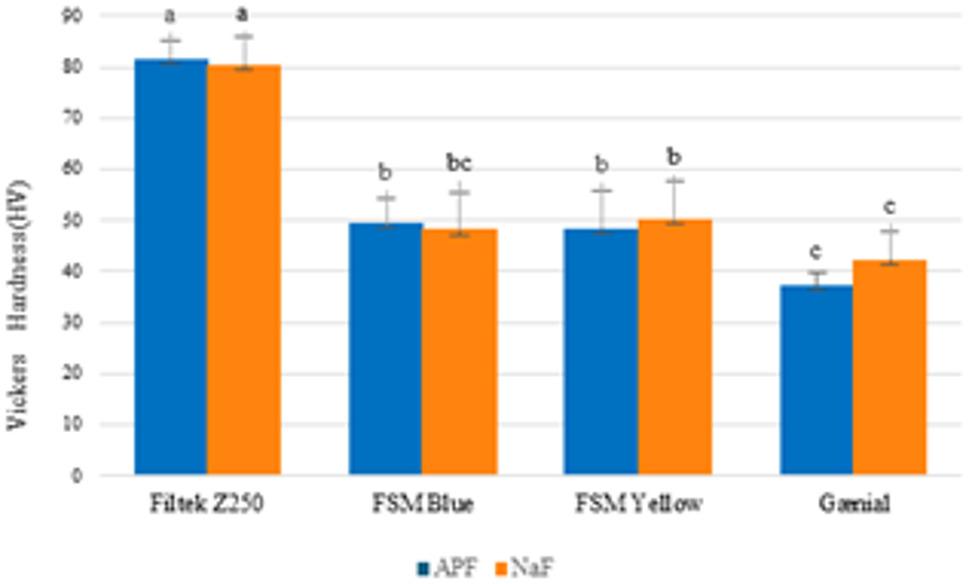
Comparison of surface microhardness (mean ± SD, HV) of composite resins after 24-hour exposure to APF and NaF gels. Bars represent mean values, and error bars indicate standard deviation. Different superscript letters indicate statistically significant differences between materials within the same condition (Tukey HSD, *p* < 0.05)

Figure 1 illustrates the comparative surface microhardness of composite resins following 4-min fluoride exposure, while Fig. 2 presents the corresponding findings following 24-h exposure. 

### SEM Analysis

**Fig. 3:**
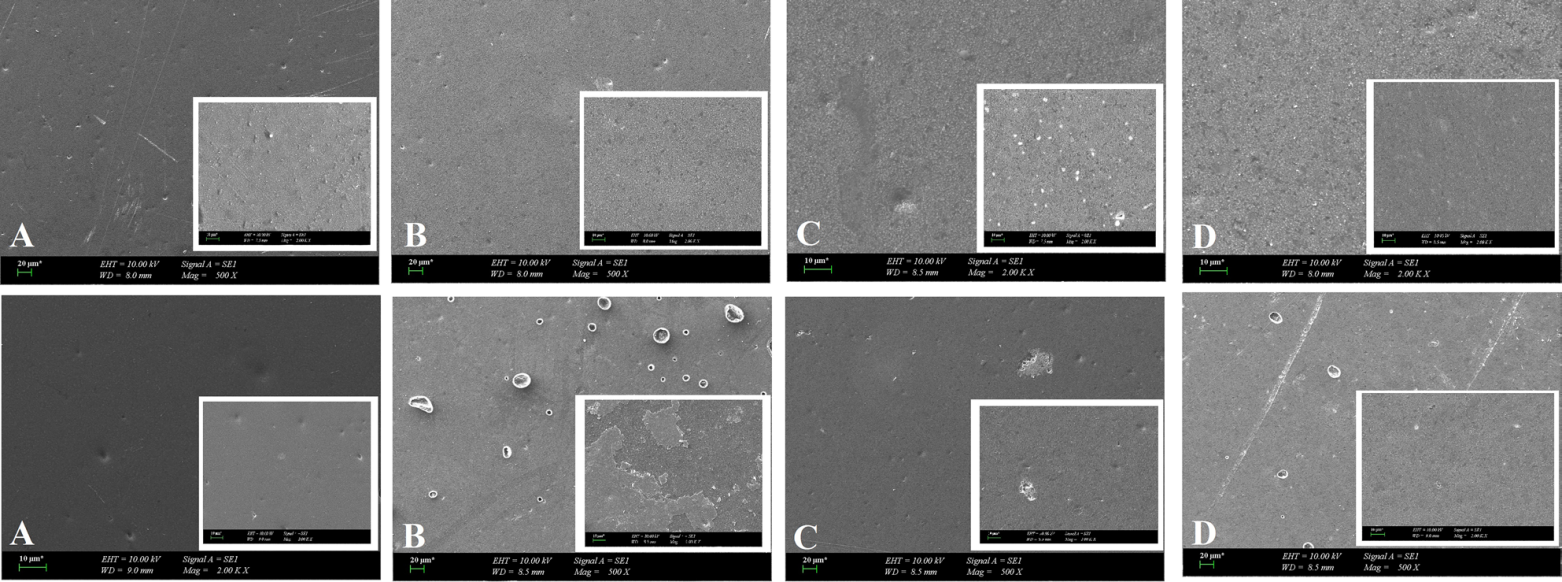
Representative SEM images of composite resin surfaces after APF exposure. Upper row: 4 min exposure: (**A**) Filtek Z250, (**B**) FSM Hi-Fill Kids Blue, (**C**) FSM Hi-Fill Kids Yellow, and (**D**) Gænial. Lower row: 24 h exposure: (**A**) Filtek Z250, (**B**) FSM Hi-Fill Kids Blue, (**C**) FSM Hi-Fill Kids Yellow, and (**D**) Gænial. Main images were obtained at ×500 magnification, and inset images represent higher-magnification views (×2000) of selected surface areas

**Fig. 4:**
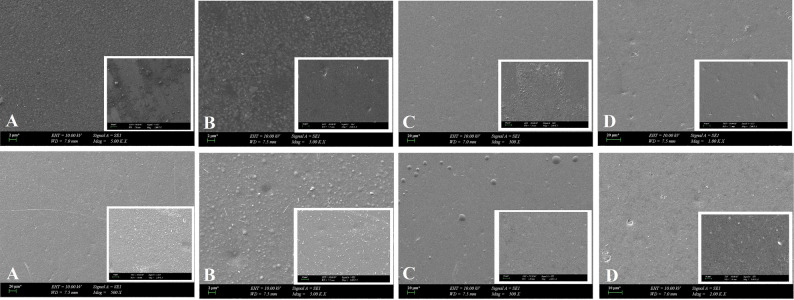
Representative SEM images of composite resin surfaces after NaF exposure. Upper row: 4 min exposure: (**A**) Filtek Z250, (**B**) FSM Hi-Fill Kids Blue, (**C**) FSM Hi-Fill Kids Yellow, and (**D**) Gænial. Lower row: 24 h exposure: (**A**) Filtek Z250, (**B**) FSM Hi-Fill Kids Blue, (**C**) FSM Hi-Fill Kids Yellow, and (**D**) Gænial. Main images were obtained at ×500 magnification, and inset images represent higher-magnification views (×2000) of selected surface areas

Representative SEM micrographs are shown in Figs. 3 and 4. APF-treated specimens generally exhibited greater surface disruption than NaF-treated specimens, including more apparent matrix irregularities and localized filler exposure. These changes were more evident after 24 h of exposure and were particularly noticeable in materials with lower microhardness values. In contrast, NaF-treated specimens tended to exhibit smoother, more uniform surface morphology. Overall, the SEM findings were consistent with the microhardness results, reinforcing the observed material-dependent response to fluoride exposure.

## Discussion

Fluoride-related surface changes varied across composite materials and were strongly influenced by both material composition and exposure duration, consistent with previous evidence highlighting the roles of fluoride formulation and pH in determining composite surface behaviour [10, 22]. Clinically, these results indicate that the selection of fluoride agents should not rely solely on their anticaries efficacy, but also on their potential impact on the integrity of restorative materials, particularly in pediatric patients who are frequently exposed to topical fluoride applications.

The greater surface alterations observed following APF exposure may be explained by its low pH, which can facilitate degradation of the resin matrix and compromise the filler–matrix interface. Acidic environments accelerate hydrolytic processes within polymer networks, leading to increased porosity and disruption of structural integrity in resin-based materials [7, 23]. Previous studies have similarly reported that acidic fluoride formulations may adversely affect composite surface properties, including increased porosity and degradation of the filler–matrix interface [7, 23]. More recent evidence further indicates that the response of resin-based materials to chemical challenges is strongly dependent on both material composition and environmental conditions [24]. In clinical practice, these findings indicate that repeated exposure to acidic fluoride agents, such as APF, may increase the risk of surface degradation in composite restorations over time, particularly under frequent preventive applications.

From a clinical perspective, these findings are particularly relevant in pediatric dentistry, where topical fluoride agents are routinely applied as part of preventive care in patients who frequently present with existing composite restorations. Repeated fluoride exposure, especially in children at high risk of dental caries, may result in cumulative effects on restorative material surfaces over time. In this context, the present findings suggest that neutral NaF formulations may represent a more conservative option when repeated fluoride applications are required in patients with composite restorations. Conversely, the use of acidic fluoride formulations, such as APF, should be carefully considered, particularly when preservation of restoration integrity is a priority.

Within this standardized surface framework, NaF was generally less disruptive to composite surfaces than APF, particularly under short-term exposure conditions that better reflect routine clinical fluoride application. This finding is clinically relevant in pediatric dentistry, where repeated topical fluoride exposure is common in preventive care [5]. The near-neutral pH of NaF may reduce direct chemical challenge to the resin matrix and filler particles, which may explain the relatively stable microhardness values observed across several materials. Nevertheless, the present findings also indicate that neutral fluoride agents should not be considered entirely inert. Under prolonged NaF exposure, subtle material-specific changes in microhardness and surface morphology were observed, suggesting that even neutral fluoride agents may influence restorative material surfaces depending on composite composition and cumulative exposure. This interpretation is consistent with previous studies indicating that the effects of preventive agents on resin composites vary according to both agent composition and restorative material formulation [3, 8].

Exposure duration was another important factor influencing material behavior. Short-term exposure, simulating professional fluoride application, was generally associated with modest surface changes, whereas prolonged exposure resulted in more pronounced material-dependent effects. Although prolonged exposure protocols do not directly replicate clinical conditions, they serve as accelerated models for evaluating the cumulative effects of repeated fluoride contact [11]. This limitation is further related to the absence of key intraoral factors such as salivary components, biofilm activity, thermal fluctuations, and mechanical loading. These factors may collectively modulate the physicochemical environment and mechanical stresses acting on restorative materials, thereby influencing their degradation behavior and clinical performance. These findings emphasize that not only the type of fluoride agent but also the frequency and duration of exposure may influence the long-term performance of composite restorations.

Time-matched distilled-water control specimens showed relatively stable hardness values over time and served as reference conditions for aqueous storage. This pattern suggests that the differences observed among the experimental groups were predominantly due to fluoride exposure and material-dependent responses, although the potential influence of aqueous storage cannot be completely ruled out. It should also be noted that distilled water does not replicate the buffering capacity of saliva or the protective effects of the acquired pellicle, which may influence material behavior under clinical conditions. These findings are consistent with previous reports indicating that neutral fluoride formulations produce limited measurable surface changes in resin composites compared with non-fluoride conditions. Arruda et al. [8] reported minimal or no significant roughness changes after neutral fluoride exposure and described SEM appearances largely comparable to those of untreated specimens, whereas APF was associated with more pronounced surface changes.

Material-dependent differences were among the most prominent findings of this study and underscore the critical role of composite formulation in resistance to fluoride-related surface changes. Among the tested materials, Filtek Z250 consistently exhibited the highest microhardness values, whereas Gænial showed the lowest values across most conditions. These differences are likely related to variations in filler loading, filler characteristics, and resin matrix composition. The differential response of materials may also be influenced by differences in monomer systems (e.g., Bis-GMA, UDMA), which affect susceptibility to acidic degradation. Variations in monomer structure, degree of cross-linking, and hydrophilicity may influence water sorption and hydrolytic degradation processes under acidic conditions, thereby modulating material performance, as supported by previous studies demonstrating the role of resin matrix composition and hydrolytic degradation in composite behavior under oral and acidic conditions [25–27].

Materials with higher filler content and more stable filler–matrix coupling are generally more resistant to chemical degradation and surface softening. This interpretation is supported by broader evidence showing that composite degradation in simulated oral environments depends on both matrix chemistry and filler characteristics [2]. In addition, chemical challenges have been shown to adversely affect the mechanical properties and surface behavior of composite resins, further supporting the role of material composition in determining resistance to degradation [28]. The superior performance of Filtek Z250 may therefore be attributed to its microhybrid filler system and relatively favorable resistance to fluoride-related surface challenge. In contrast, materials with lower filler loading or different matrix compositions may be more susceptible to surface alterations under chemical challenge. These observations reinforce that the composite response to chemical challenge is governed by intrinsic material properties. The inclusion of both microhybrid and nanohybrid materials was intentional to reflect the diversity of composite resins used in pediatric clinical practice; however, these structural differences should also be considered a potential confounding factor when interpreting fluoride-related effects.

The comparable responses observed between the two FSM shade variants suggest that differences in pigmentation were unlikely to significantly influence fluoride-related surface behavior under the present conditions. Rather, the underlying resin-filler formulation appears to be the primary determinant of material performance. These findings further support the interpretation that compositional factors, rather than color differences, govern the susceptibility of composite materials to fluoride-induced surface changes.

The SEM observations were in qualitative agreement with the microhardness findings. Specimens exposed to APF generally exhibited more pronounced surface disruption, including matrix irregularities and localized filler exposure, particularly after prolonged exposure. In contrast, NaF-treated specimens tended to exhibit smoother, more uniform surface morphology. These changes were more evident after prolonged exposure, particularly in materials with lower microhardness values. Although the SEM analysis was qualitative and based on a limited number of representative specimens, the observations should be interpreted as exploratory rather than definitive evidence. Nevertheless, the consistency between mechanical and morphological findings supports the interpretation that acidic fluoride exposure may induce more evident surface alterations in susceptible composite systems, consistent with previous SEM-based studies on the effects of topical fluoride on resin-based restorations [7, 23]. This correspondence further supports the robustness of the observed material-dependent responses.

In the present study, all specimens were polymerized against Mylar strips to ensure standardized surface conditions and minimize variability related to finishing and polishing procedures. This approach is consistent with previous microhardness studies, in which Mylar strip surfaces are used to provide controlled conditions for comparing materials and treatments [29]. At the same time, curing against Mylar is known to produce a resin-rich superficial layer that may differ from clinically finished, filler-exposed surfaces and may therefore influence surface hardness outcomes [30]. Accordingly, some of the fluoride-related changes observed in the present study, particularly under acidic conditions, may be attributable to the greater susceptibility of this superficial layer to matrix softening or degradation. Nevertheless, because all specimens were prepared under identical standardized conditions, the comparative interpretation of material- and exposure-related differences remains valid.

Several considerations should be taken into account when interpreting the present findings. The standardized in vitro design allowed controlled evaluation of fluoride-related surface changes; however, the absence of salivary components, biofilm activity, thermal fluctuations, and mechanical loading may influence material behavior differently under clinical conditions. Distilled water was used to standardize storage conditions and isolate the chemical effects of fluoride; nevertheless, it does not replicate the buffering capacity and protective functions of saliva. In addition, the prolonged exposure protocol should be interpreted as an accelerated laboratory model of cumulative fluoride contact, providing an approximation of cumulative effects rather than a direct representation of intraoral clinical conditions. SEM assessments were qualitative rather than quantitative, representative specimens, and should therefore be interpreted as exploratory rather than definitive evidence of surface alterations. Despite these limitations, the study has several strengths, including its controlled factorial design, the inclusion of multiple composite materials relevant to pediatric dentistry, and the combined use of quantitative microhardness testing and qualitative SEM assessment to evaluate fluoride-related surface changes. Further in situ and clinical studies are needed to better define the long-term interaction between fluoride agents and composite restorations. These findings also highlight a potential direction for material-level optimization. Composite resins may benefit from improved filler stability, filler–matrix coupling, and resistance of the resin matrix to hydrolytic degradation in fluoride-rich environments, which may help preserve surface integrity during repeated preventive care.

## Conclusions

Topical fluoride gels affected the surface microhardness of composite resins in a material-dependent manner, with APF causing greater reductions than NaF, particularly under prolonged exposure. From a clinical perspective, when repeated fluoride applications are required, neutral fluoride formulations, such as NaF, should be preferred over acidic agents, such as APF, to minimize the risk of surface degradation. These findings also underscore the importance of material selection for preserving the long-term integrity of composite restorations.

## Data Availability

The datasets generated and/or analyzed during the current study are available from the corresponding author on reasonable request.
